# Unique characteristics and outcomes of therapy-related acute lymphoblastic leukemia following treatment for multiple myeloma

**DOI:** 10.1038/s41408-022-00680-y

**Published:** 2022-06-01

**Authors:** Ricardo D. Parrondo, Zaid Abdel Rahman, Michael G. Heckman, Mikolaj Wieczorek, Liuyan Jiang, Hassan B. Alkhateeb, Mark R. Litzow, Patricia Greipp, Taimur Sher, Leif Bergsagel, Rafael Fonseca, Vivek Roy, Angela Dispenzieri, Mohamed A. Kharfan-Dabaja, Hemant S. Murthy, Sikander Ailawadhi, James M. Foran

**Affiliations:** 1 Division of Hematology-Oncology and Blood and Marrow Transplantation Program, Mayo Clinic, Jacksonville, FL USA; 2 Stem Cell Transplantation and Cellular Therapies, MD Anderson Cancer Center, Houston, TX USA; 3 Division of Clinical Trials and Biostatistics, Mayo Clinic, Jacksonville, FL USA; 4 Department of Laboratory Medicine and Pathology, Mayo Clinic, Jacksonville, FL USA; 5 Division of Hematology, Mayo Clinic, Rochester, MN USA; 6 Department of Laboratory Medicine and Pathology, Mayo Clinic, Rochester, MN USA; 7 Division of Hematology-Oncology, Mayo Clinic, Phoenix, AZ USA

**Keywords:** Acute lymphocytic leukaemia, Myeloma

Therapy-related acute lymphoblastic leukemia (tr-ALL) is an important secondary primary malignancy (SPM) that has recently been appreciated and has an estimated incidence of 3–9% of ALL cases [1–4]. Three large phase III clinical trials have demonstrated a significant increased risk of SPM associated with lenalidomide maintenance following therapy with high dose melphalan and autologous hematopoietic cell transplantation (AHCT) in patients with multiple myeloma (MM) with an SPM incidence of 8–17% and with 4–17% of those malignancies being hematologic malignancies [5–7]. The number of trALL cases in these trials has not been reported. Little is known about the characteristics of trALL in patients with MM compared to patients who had other malignancies prior to the development of trALL. We define tALL as ALL that developed after any prior exposure to cytotoxic chemotherapy and/or radiation for another malignancy, and herein, we report a comparative analysis of characteristics and outcomes of patients with trALL with and without MM from the Mayo Clinic Cancer Center (MCCC).

We performed a systematic search in the 3-site MCCC registry and included all patients diagnosed with ALL and who received at least 1 cycle of ALL-directed therapy and/or underwent allogeneic transplantation (AlloHCT) for ALL between 2007 and 2020. All cases of trALL in this series are ALL of B cell lineage. Comparisons of characteristics between trALL patients with and without MM were made using a Wilcoxon rank sum test (continuous variables) or Fisher’s exact test (categorical variables). Comparisons of outcomes between patients with and without MM were made using unadjusted logistic regression (MRD and complete remission) and Cox regression (death, relapse, NRM) models. In Cox models, the cause-specific hazard of the given outcome was modeled, and cumulative incidences were estimated while accounting for the competing risk of death when relevant [8]. Statistical analyses were performed using R Statistical Software.

Seventy trALL patients (14 MM [20%], 56 non-MM [80%]) were identified during the study period. The median age at trALL diagnosis was 63.9 (range 47–71) years for MM patients and 63.1 (range 18.2–84) years for non-MM patients. Baseline patient characteristics are shown in Table 1. The median follow-up from trALL diagnosis was 1.42 years (range 0.02–10.6 years). The most common prior non-MM malignancies among trALL patients were breast (28.6%), lymphoma (17.9%), myeloid (17.9%), and GU/GYN (14.3%). Among the 14 MM patients, 100% (*n* = 10) had ISS-I disease, 8 (57%) had IgG K MM, 1 (*n* = 10) had high risk cytogenetics. Nine (64.2%) received lenalidomide-based induction; 3 had lenalidomide-dexamethasone, 4 had bortezomib-lenalidomide-dexamethasone, 2 had lenalidomide combined with another agent. Four patients (28.6%) received cyclophosphamide-bortezomib-dexamethasone induction. Eight patients (57.1%) had prior AHCT.

**Table 1:** Characteristics of trALL patients at trALL diagnosis and initial trALL therapies.

		Median (minimum, maximum) or No. (%) of patients		
Variable	*N*	MM trALL patients (*N* = 14)	Non-MM trALL patients (*N* = 56)	*P*-value
**Age at diagnosis (years)**	70	63.9 (46.6, 70.9)	63.1 (18.2, 83.5)	0.79
**Sex (Male)**	70	4 (28.6%)	19 (33.9%)	1.00
**Race**	65			0.72
**White**		13 (92.9%)	47 (92.2%)	
**Black**		1 (7.1%)	1 (2.0%)	
**Asian**		0 (0.0%)	2 (3.9%)	
**Other**		0 (0.0%)	1 (2.0%)	
**Ethnicity (Hispanic or Latino)**	58	0 (0.0%)	2 (4.5%)	1.00
**WBC**	**49**	**2.0 (0.9, 12.0)**	**5.8 (0.5, 135.0)**	**0.005**
**Hemoglobin**	41	9.4 (6.3, 12.8)	9.8 (5.4, 14.8)	0.63
**Platelets**	42	38.0 (16.0, 261.0)	89.0 (8.0, 313.0)	0.20
**Cytogenetic group**	70			
**t(9;22) BCR/ABL1**		**0 (0.0%)**	**20 (35.7%)**	**0.008**
**MLL/KMT2A rearrangement**		0 (0.0%)	5 (8.9%)	0.25
**t(1;19) TCF3/PBX1**		0 (0.0%)	2 (3.6%)	0.47
Ho-Tri*		**6 (42.9%)**	**7 (12.5%)**	**0.009**
HeH**		2 (14.3%)	2 (3.6%)	0.12
**Normal Karyotype** + **FISH**		1 (7.1%)	4 (7.1%)	1.00
**Other**		2 (14.3%)	12 (21.4%)	0.55
**p16/CDKN2A**		1 (7.1%)	1 (1.8%)	0.28
**Complex - Moorman’s**		2 (14.3%)	3 (5.4%)	0.25
**Induction chemo**	70			0.13
**HyperCVAD**		10 (71.4%)	29 (51.8%)	
**Pediatric**		0 (0.0%)	2 (3.6%)	
**ECOG regimens**		3 (21.4%)	6 (10.7%)	
**Others**		1 (7.1%)	19 (33.9%)	
**CNS involvement**	70	0 (0.0%)	9 (16.1%)	0.19
**Transplant**	70	10 (71.4%)	24 (42.9%)	0.075
**ALL status at transplant**	34			0.078
**CR1**		10 (100.0%)	17 (70.8%)	
**≥CR2**		0 (0.0%)	7 (29.2%)	
**Graft type**	34			1.00
**BM**		0 (0.0%)	2 (8.3%)	
**PB**		10 (100.0%)	21 (87.5%)	
**UC**		0 (0.0%)	1 (4.2%)	
**Donor type**	34			0.094
**Matched related**		4 (40.0%)	11 (45.8%)	
**Haploidentical**		3 (30.0%)	1 (4.2%)	
**Matched unrelated**		3 (30.0%)	12 (50.0%)	
**Conditioning regimen**	34			0.13
**MAC**		2 (20.0%)	13 (54.2%)	
**NMA/RIC**		8 (80.0%)	11 (45.8%)	
**Previous malignancy**	70			
**Breast**		0 (0.0%)	16 (28.6%)	
**GI**		0 (0.0%)	3 (5.4%)	
**GU/GYN**		0 (0.0%)	8 (14.3%)	
**Lung**		0 (0.0%)	2 (3.6%)	
**H&N**		0 (0.0%)	1 (1.8%)	
**Thyroid**		0 (0.0%)	2 (3.6%)	
**HD**		0 (0.0%)	2 (3.6%)	
**NHL**		0 (0.0%)	10 (17.9%)	
**Multiple myeloma**		14 (100.0%)	0 (0.0%)	
**Myeloid**		0 (0.0%)	10 (17.9%)	
**Other**		0 (0.0%)	2 (3.6%)	
**Chemotherapy**		14 (100.0%)	46 (82.1%)	0.19
**Radiation therapy**		**1 (7.1%)**	**25 (44.6%)**	**0.012**
**Chemotherapy and radiation therapy**		1 (7.1%)	15 (26.8%)	0.16
**Time to development of therapy-related ALL (years)**		6 (2, 14)	5 (1, 29)	0.87

Bold values identify statistical significance (*P* < 0.05)

*P*-values result from a Wilcoxon rank sum test (continuous variables) or Fisher’s exact test (categorical variables).

*Hypodiploidy/near triploidy (Ho-Tri)

**Hyperdiploidy (HeH)

The median time to develop trALL following AHCT for MM was 46.3 months (m), (range 20.4–67.6). Twelve patients (85.7%) received lenalidomide maintenance for a median duration of 53 m (range 16.9–121). The median time to develop trALL after initiation of lenalidomide maintenance was 61.2 m (range 16.9–123.4). Ten (71.4%) patients were receiving lenalidomide at the time of their trALL diagnosis. There was a statistically significant difference in median time to development of trALL based on previous malignancy diagnosis with GU/GYN at 9 years (longest time), myeloid at 3 years (shortest time), and MM at 6 years (*P* = 0.018). In comparison to non-MM patients, MM patients had a significantly lower WBC at diagnosis (Median: 2.0 vs. 5.8, *P* = 0.005), no t(9;22)/BCR/ABL1 mutation (0.0% vs. 35.7%, *P* = 0.008), a higher frequency of hypodiploid/near-triploid (Ho-Tri) cytogenetics (42.9% vs. 12.5%, *P* = 0.009), and a lower frequency of prior radiation therapy (7.1% vs. 44.6%, *P* = 0.012). The most common trALL induction regimen received was HyperCVAD which was used in 71.4% of MM patients and 51.8% of non-MM patients. No trALL in MM patients had a Philadelphia-like phenotype. In the overall patient cohort, 56 (80%) patients achieved a complete response after induction and 60% (15/25) were minimal residual disease (MRD) positive (+) after induction. Patients with MM had a significantly higher likelihood of being MRD negative after induction (Odds ratio = 0.09, *P* = 0.015). Though not quite statistically significant, MM patients were more likely to undergo AlloHCT for trALL (71.4% vs. 42.9%, *P* = 0.075) and were more commonly in complete response (CR) at transplant (100.0% vs. 70.8%, *P* = 0.078). Overall, 24.3% (*n* = 17) of patients relapsed after induction therapy for trALL; 39 (55.5%) died after trALL diagnosis including 42.9% (15/35) who died after AlloHCT. Although patients with MM tended to have better outcomes compared to non-MM trALL patients in terms of overall survival after trALL diagnosis (Fig. 1A), survival after AlloHCT (Fig. 1B), relapse rate after trALL diagnosis, and non-relapse mortality after AlloHCT, none of these findings approached significance in this small cohort (Fig. 1C).

**Fig. 1:**
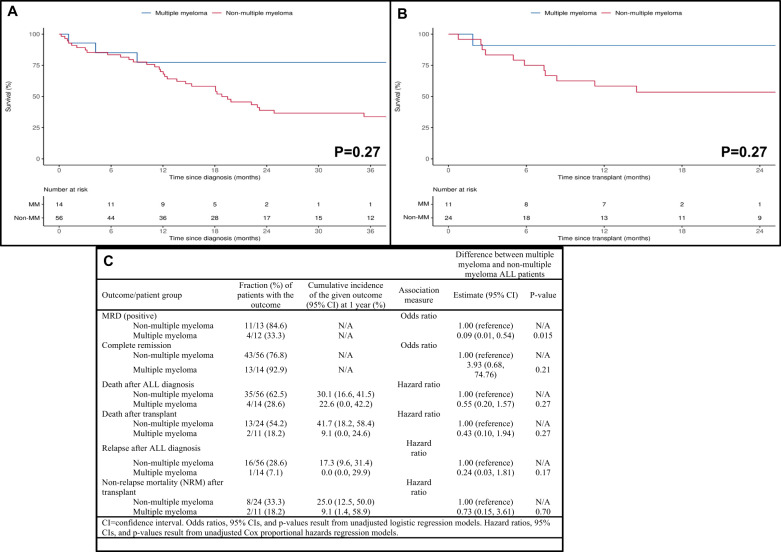
Comparison of outcomes between multiple myeloma ALL patients and non-multiple myeloma ALL patients. **A** Survival after ALL diagnosis for multiple myeloma (MM) and non-multiple myeloma (non-MM) patients. **B** Survival after AlloHCT for multiple myeloma (MM) and non-multiple myeloma (non-MM) patients **C** Comparison of outcomes between multiple myeloma ALL patients and non-multiple myeloma ALL patients.

We report one of the few studies evaluating the characteristics of trALL in patients with antecedent MM. A previous study which evaluated 13 patients with trALL after therapy for MM with whole exome sequencing reported that the two malignancies arise from different clones and almost all patients had received AHCT and lenalidomide maintenance, like our patient population [9]. Compared to non-MM trALL, MM-trALL is associated with Ho-Tri cytogenetics and is BCR/ABL1 negative in our cohort of patients. While previous characterizations of trALL report that adverse cytogenetics are common [10], in our study, patients with prior MM who developed trALL tended to have more adverse cytogenetic features compared to non-MM trALL. Previous reports have shown that BCR/ABL1 mutations can occur in trALL, however, none of the MM patients with trALL had BCR/ABL1 mutations in our study [10, 11]. AlloHCT can produce comparable long-term survival outcomes in patients with trALL compared to de novo ALL [10, 12], however, in our study, AlloHCT did not improve survival outcomes of patients with trALL and antecedent MM compared to other malignancies despite MM patients being more likely to be in CR after trALL induction and MRD negative prior to AlloHCT.

Limitations of our study include the retrospective design which introduces biases into the data collection and the relatively small number of patients with trALL with antecedent MM which resulted in a lack of power to detect differences between groups. Next-generation sequencing (NGS) of bone marrow aspirates of 3 patients with antecedent chronic lymphocytic leukemia who developed trALL while on lenalidomide maintenance revealed an IKFZ1 mutation in one of the patients [13]. Lenalidomide is known to induce proteasomal degradation of the transcription factor Ikaros which is encoded by IKFZ1 and mutations or deletions IKZF1 are frequent driver lesions in ALL and promote leukemogenesis [14]. Thus, perhaps lenalidomide-induced alterations in IKZF1 may be implicated in the development of trALL. Further research on trALL in MM patients is merited with a particular focus on comparative NGS to identify key driver lesions in this dreaded complication of MM therapy.

## Data Availability

The datasets generated during and/or analyzed during the current study are not publicly available due to them containing patient personal health information but are available from the corresponding author on reasonable request for a de-identified data set.
